# Iron homeostasis at the interface of nutrition, infection, and iron supplementation

**DOI:** 10.3389/fnut.2026.1943495

**Published:** 2026-10-12

**Authors:** Özge Mert, Seray Saday, Aybala Temel

**Affiliations:** 1 Institute of Health Sciences, Izmir Katip Celebi University, Izmir, Türkiye; 2 Department of Pharmaceutical Microbiology, Faculty of Pharmacy, Izmir Katip Celebi University, Izmir, Türkiye

**Keywords:** ferroptosis, hepcidin, infection, iron homeostasis, iron supplements, nutrition

## Abstract

Iron is an essential micronutrient that plays a central role in oxygen transport, mitochondrial respiration, DNA synthesis, immune regulation, and numerous enzymatic reactions. Because both iron deficiency and iron overload adversely affect health, systemic and cellular iron homeostasis is tightly regulated through coordinated mechanisms involving intestinal absorption, macrophage-mediated recycling, iron storage, and the hepcidin–ferroportin axis. During infection and inflammation, the host restricts iron availability through nutritional immunity, thereby limiting pathogen proliferation while influencing immune cell function and iron distribution. This review provides an integrated overview of iron homeostasis by linking nutritional factors, inflammatory regulation, infection dynamics, and iron supplementation strategies. The molecular mechanisms governing intestinal iron absorption are summarized together with dietary factors influencing iron bioavailability, including vitamin C, phytates, polyphenols, calcium, dietary fiber, and dietary patterns. The central role of the hepcidin–ferroportin axis in inflammation-induced hypoferremia and its clinical implications for celiac disease, inflammatory bowel disease, aging, and pregnancy are also discussed. Particular emphasis is placed on the dual role of iron in host defense, highlighting how nutritional immunity restricts extracellular pathogens while exerting distinct effects on intracellular microorganisms and adaptive immune responses. The benefits and limitations of iron supplementation are critically evaluated, emphasizing gastrointestinal tolerance, oxidative stress, gut microbiota alterations, dysbiosis, and infection risk. Overall, iron metabolism should be regarded as a dynamic network linking nutrition, immune regulation, microbial ecology, and clinical therapeutics. A comprehensive understanding of these interactions may facilitate personalized iron replacement strategies that effectively correct iron deficiency while preserving intestinal homeostasis and antimicrobial defense.

## 1 Introduction

Iron is a trace element that acts as a cofactor in many biological processes, such as hemoglobin and myoglobin synthesis, oxygen transport, mitochondrial respiration, DNA and protein synthesis, enzyme production and activation, and the regulation of the immune response (1). Iron is a key component of hemoglobin in red blood cells and myoglobin in muscles, and it exists in two forms in the body. The first form is found in the blood, is transported by the transferrin carrier protein, and is used for hemoglobin synthesis. The second form is the storage form of iron, which is bound to the proteins ferritin and hemosiderin. More than half of the iron in the body is found in hemoglobin; 25% is stored in the liver, spleen, and bone marrow; and 15% is bound to myoglobin and other enzymes (2).

Iron plays a critical role in many enzymatic and metabolic processes in the body. Therefore, significant changes, disruptions, and diseases in physiological processes may arise as a result of iron deficiency or excess. Iron’s ability to catalyze redox reactions and the production of reactive oxygen species may lead to toxic effects in the body (3, 4). High iron levels have been linked to neurodegenerative diseases, heart disease, diabetes, kidney and liver diseases, and other conditions over the long term. Although no specific mechanism regulating the excretion of excess iron from the body is known in mammals, it has been established that iron loss occurs through processes such as bleeding, intestinal mucosal renewal, and the shedding of dead skin cells (2). Conversely, low iron levels can also lead to the development of anemia and related disorders in the long term (4). For this reason, iron homeostasis in mammals is critically important for the organism’s health and is maintained under a strict regulatory mechanism (3).

The average adult contains approximately 3–4 grams of iron, and about 2–3 grams of this is found in erythrocyte hemoglobin. In blood plasma, however, there are only 2–4 mg, which are transported between tissues bound to transferrin. Plasma iron is renewed every few hours, and approximately 20–25 mg of iron passes through it daily. The majority of plasma iron does not come from iron absorbed from the diet through the intestines, but rather from aged red blood cells recycled by macrophages. The average lifespan of red blood cells is 120 days. For this reason, 0.8% of the iron in red blood cells (15–25 mg/day) must be recycled every day. Aged red blood cells are phagocytosed by macrophages, and the iron in hemoglobin is not lost. Macrophages transfer the recovered iron to transferrin via ferroportin. Transferrin, in turn, transports this iron to the bone marrow, ensuring it is reused for erythropoiesis. While the majority of the 20–25 mg of iron required daily for erythropoiesis is provided by this recycling process, the iron absorbed from the diet amounts to only 1–2 mg per day and is used solely to compensate for physiological iron losses (5).

## 2 Iron and nutrition

### 2.1 Iron absorption in gastrointestinal system

Iron obtained from the diet comes in two forms: non-heme iron, found in animal and plant foods, is mostly in the ferric (Fe^3+^) form and has low bioavailability. In contrast, heme iron obtained from animal foods has higher absorption and bioavailability. Furthermore, it has been reported that heme iron supports the absorption of non-heme iron through a mechanism known as the “meat factor” (6).

Iron absorption in the human body occurs in the duodenum and upper jejunum. Iron absorption in the body is completed in three basic stages: passage from the intestinal lumen into enterocytes, regulation within the enterocytes, and transport from the basolateral membrane into the bloodstream (Figure 1). Dietary iron that reaches the intestinal lumen is present in two forms: non-heme (Fe^3+^) and heme iron. Dietary iron is predominantly present in the (Fe^3+^) form. However, the DMT1 transporter protein can only transport iron from the intestinal lumen into enterocytes in the (Fe^2+^) form. For this reason, iron is converted from the (Fe^3+^) form to the (Fe^2+^) form by ferric reductase enzymes on the apical surface of enterocytes. One of the enzymes most actively involved in this stage is Dcytb (duodenal cytochrome b), which is secreted by duodenal intestinal cells. At the end of the process, the iron converted to the (Fe^2+^) form is transported into the enterocyte via the DMT1 transporter protein located in the apical membrane (7).

**Figure 1:**
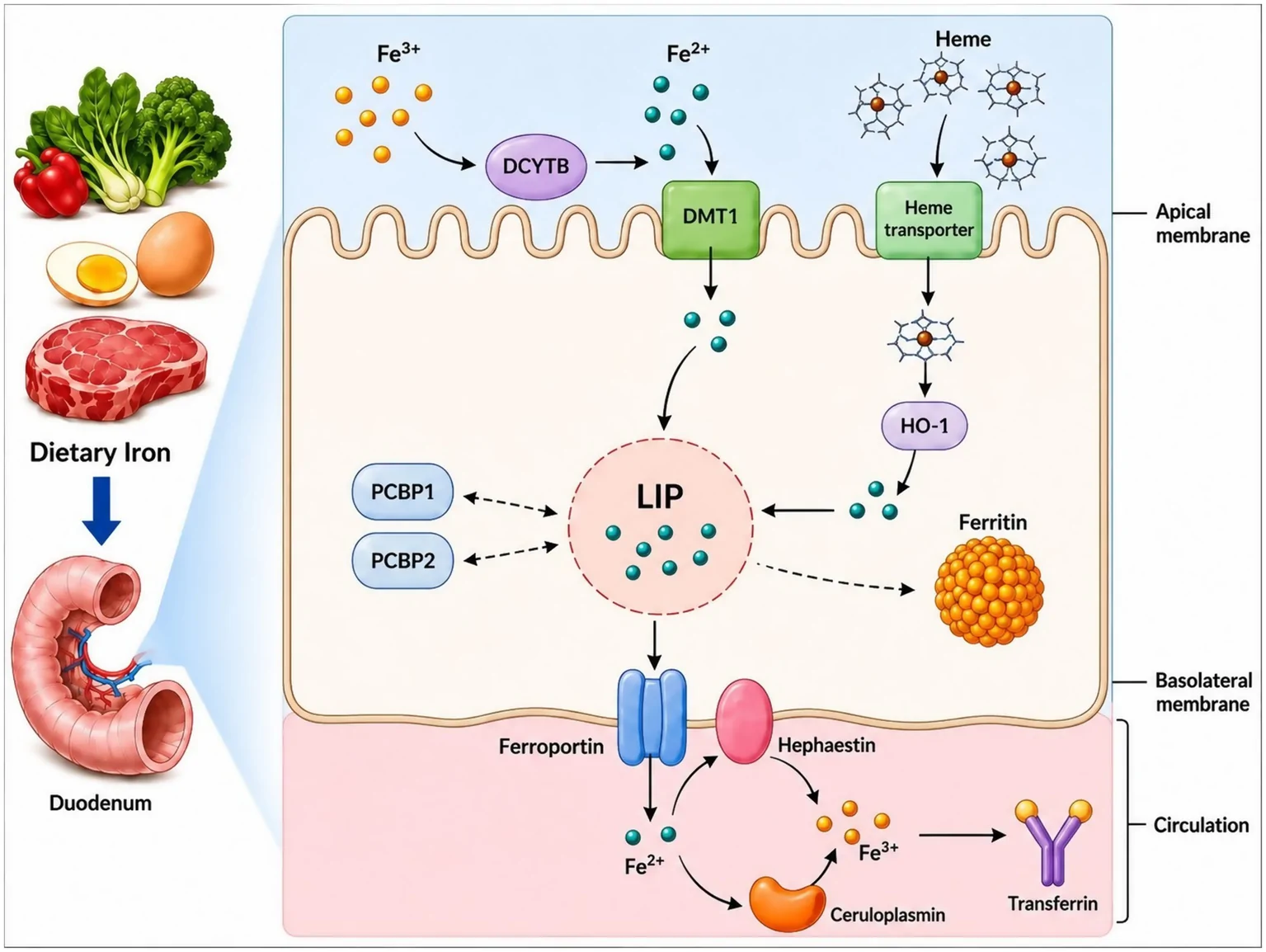
Molecular mechanism of intestinal iron absorption and intracellular iron trafficking. Iron obtained from the diet reaches the intestinal lumen in two forms: non-heme iron (Fe^3+^) and heme iron. Non-heme Fe^3+^ is converted to the Fe^2+^ form by the DCYTB ferric reductase on the cell membrane so that it can be transported into the intestinal cell, and is then taken up into the cell by DMT1. Heme iron, on the other hand, is taken up into the cell via a mechanism that has not yet been fully elucidated; it is broken down intracellularly by heme oxygenase-1 (HO-1), releasing the Fe^2+^ within its structure. The iron from these two pathways is pooled in the labile iron pool (LIP). From there, iron is stored by ferritin, transported to intracellular target proteins via chaperone proteins such as PCBP1 and PCBP2, or transported into the bloodstream via ferroportin on the cell’s basolateral membrane. Following transport, Fe^2+^ is oxidized to Fe^3+^ by hephaestin or ceruloplasmin, allowing it to bind to plasma transferrin and enter the circulation.

The uptake of heme iron—found in meat, poultry, and seafood—into enterocytes, however, occurs via a mechanism that differs from that of non-heme iron and has not yet been fully elucidated. Heme iron is broken down by the HO-1 (heme oxygenase 1) enzyme within the enterocyte, separating into the heme component and (Fe^2+^) (8). (Fe^2+^) iron that reaches the enterocyte can follow three different pathways within the cell. It can be utilized within the cell, stored in the form of ferritin, or exported out of the cell via ferroportin. Which of these three pathways iron will follow is largely determined by the body’s iron requirements. (Fe^2+^) is transported into the bloodstream via the ferroportin channel located on the enterocyte (5). However, the (Fe^2+^) form cannot remain stable in the blood and must be converted to the (Fe^3+^) form. There are two different enzymes with the same function involved in this conversion. Iron is converted from the (Fe^2+^) form to the (Fe^3+^) form by the hephaestin enzyme, which is produced by intestinal cells and located on the basolateral membrane of enterocytes, and by the ceruloplasmin enzyme, which is produced by the liver and enters the bloodstream. As a result, iron in the (Fe^3+^) form binds to the transferrin protein and is transported in the blood in a more stable state (9).

### 2.2 Main factors affecting Iron absorption

Iron absorption can vary depending on the presence of different foods and dietary components. While vitamin C and organic acids have a positive effect on iron absorption, study results indicate that components such as phytic acid, polyphenols, tannins, calcium, and oxalic acid can inhibit iron absorption (10).

#### 2.2.1 Vitamin C

Vitamin C, also known as ascorbic acid, is a water-soluble vitamin that the body cannot produce and must therefore be obtained through food. As a powerful antioxidant, vitamin C plays a role in important metabolic processes in the human body, primarily by strengthening the immune system, promoting wound healing, and supporting collagen production. In addition, there is a strong and clinically significant relationship between vitamin C and iron. It is frequently recommended in combination with iron therapy because it enhances the absorption of plant-based iron (non-heme iron). When foods containing vitamin C (ascorbate) are consumed, vitamin C works in conjunction with the Dcytb enzyme to support the reduction of the (Fe^3+^) form to the (Fe^2+^) form, thereby contributing to increased absorption of non-heme iron (7). However, a randomized controlled trial conducted in 2020 involving patients with iron-deficiency anemia found that vitamin C supplementation, in addition to oral iron supplementation, did not result in a significant improvement in iron levels such as ferritin and hemoglobin (11). Although vitamin C supports iron absorption, the question of whether it contributes to a significant improvement in iron levels in patients with iron-deficiency anemia requires further clinical research.

#### 2.2.2 Plant-based diet inhibitors

Although a plant-based diet is rich in fiber, vitamins, minerals, and bioactive compounds, some of its natural components can reduce the bioavailability of iron. The most important of these are phytates (phytic acid) and polyphenols. While these compounds primarily affect the absorption of plant-based non-heme iron, they have a much more limited effect on heme iron from animal sources. Phytates and polyphenols are among the primary plant-based dietary inhibitors that reduce iron absorption by forming complexes with dietary iron (10). Phytate is a bioactive compound found primarily in grains, legumes, and oilseeds that reduces the bioavailability of many minerals. It has been demonstrated that phytates can reduce iron absorption in a dose-dependent manner. It has been observed that the phytic acid content of grain flours and whole-wheat bread can be reduced by 47–100%, depending on the grain composition, through lactic acid fermentation. One study evaluated oat porridge, which is rich in phytic acid. It was found that fermented oats provided greater non-heme iron absorption compared to unfermented oats. However, it has been reported that the inhibitory effect of phytate can be eliminated by adding sufficient amounts of ascorbic acid to the diet.

Polyphenols are another broad group of compounds found naturally in plants that exhibit strong antioxidant properties. Tea, coffee, cocoa, red wine, certain fruits, vegetables, and spices are significant sources of polyphenols (10). Polyphenols reduce iron absorption in the intestine, particularly by forming insoluble complexes with non-heme iron. This effect varies depending on the type and amount of polyphenol (10). Black tea, which is particularly rich in tannins, can significantly inhibit iron absorption. Similarly, chlorogenic acids found in coffee also exhibit an absorption-inhibiting effect. In a 2021 study by Lazrak et al. involving a total of 46 women—divided into healthy and anemic groups—participants were given bread enriched with NaFeEDTA. This enriched bread was first served to the participants with water and then with tea. As a result, it was found that tea polyphenols reduced iron absorption by 85% in both anemic and healthy women (12). In addition, it has been reported that when ascorbic acid is added to the diet in sufficient amounts, the inhibitory effect of polyphenols is partially eliminated (13).

#### 2.2.3 Other dietary factors: calcium, protein sources, dietary fiber, and oxalates

##### 2.2.3.1 Calcium

The foods richest in calcium are milk and dairy products. Certain foods, such as spinach, broccoli, leafy green vegetables, and soybeans, also contain significant amounts of calcium (10). It is known that calcium can inhibit iron absorption, particularly at high doses. Unlike other inhibitors, calcium has been shown to significantly inhibit the absorption of both heme and non-heme iron; however, the exact mechanism underlying this effect has not yet been fully elucidated (7).

##### 2.2.3.2 Protein sources

It has been established that, depending on their source, proteins can either enhance or inhibit iron absorption (10). While the ‘meat factor’ found in meat and poultry has an enhancing effect on iron absorption (6), it has been reported that phosvitin in egg yolk may reduce the bioavailability of non-heme iron due to its high affinity for iron (14).

##### 2.2.3.3 Dietary fiber

Dietary fiber is classified as soluble or insoluble fiber based on its solubility in water. Studies examining the effect of dietary fiber on iron absorption have reported conflicting results. It has been suggested that soluble fibers (pectin, β-glucan, guar gum, etc.) may increase the viscosity of the chyme by forming a gel in the intestinal lumen and, as a result, may affect the absorption of nutrients. However, clinical findings demonstrating the effect of this mechanism on iron absorption are inconsistent (164, 167). Nevertheless, there are also studies suggesting that soluble fiber may support iron absorption by fermenting in the gut to produce short-chain fatty acids, lowering colonic pH, and increasing iron solubility. However, human studies have not yielded consistent findings indicating that soluble fiber supports iron absorption (13). Studies on insoluble fiber (cellulose, hemicellulose, lignin), on the other hand, indicate that insoluble fiber—particularly that found in whole grains—and components such as phytic acid present in these foods reduce the absorption of non-heme iron, while the effect of soluble fiber is more limited (6). However, the current findings on this topic are inconsistent, and more research is needed to examine the effects of soluble and insoluble dietary fiber on iron bioavailability.

##### 2.2.3.4 Oxalates

Oxalate and oxalic acid are found in foods such as spinach and purslane. A diet rich in oxalate-rich foods can, in the long term, lead to health problems such as urinary and bladder stones and hyperoxaluria. Although it is known that oxalic acid inhibits the absorption of calcium and zinc, its effect on iron absorption is controversial. A common hypothesis suggests that oxalate inhibits iron absorption because iron oxalate is poorly soluble in water. However, recent studies indicate that oxalic acid has no significant inhibitory effect on iron absorption (Milman NT., 2020).

### 2.3 Dietary patterns and the risk of anemia

Dietary patterns are one of the factors that play a significant role in determining iron absorption and the body’s iron status (2). While the diets of plant-based eaters predominantly contain non-heme iron, the diets of omnivores include both heme and non-heme iron, and these forms of iron vary in terms of bioavailability (15). While heme iron from meat, poultry, and seafood is absorbed at rates of 25–30%, non-heme iron from grains, legumes, and vegetables is absorbed at rates of 3–5%. This is because heme iron is less affected by dietary inhibitors such as phytates, minerals, and ascorbic acid than non-heme iron (6). Due to this difference in absorption, while iron is absorbed at a rate of 5–12% in vegetarians, this rate rises to 14–18% in individuals following a mixed diet (2, 13). It has been found that, due to the efficient absorption of heme iron, individuals who consume meat have higher serum ferritin levels—an important indicator of iron stores—compared to vegetarians (6). Furthermore, in populations that consume meat, although heme iron accounts for only 10–15% of total iron, it constitutes 40% of the iron that is absorbed and utilized (2).

In recent years, there has been a growing interest in plant-based diets even among healthy individuals in society. In a plant-based diet, the iron absorbed by the body is mostly in the form of non-heme iron, which has lower bioavailability. This can contribute to the risk of iron deficiency in people following a plant-based diet (15). Studies have shown that serum ferritin levels tend to be lower in vegan and vegetarian individuals compared to omnivores (16). However, low ferritin levels do not always lead to a dramatic decrease in hemoglobin concentrations or the development of anemia (17). Recent clinical studies have shown that, in some cases, vegan and vegetarian individuals may consume higher amounts of dietary iron than omnivores; however, due to differences in iron form and absorption, iron levels in vegan and vegetarian individuals may still be lower (16). For this reason, a vegan or vegetarian diet cannot be directly linked to anemia; however, close monitoring of serum ferritin and iron stores in these individuals is crucial.

## 3 Iron metabolism and inflammation

### 3.1 Inflammation, hypoferremia, and the hepcidin-ferroportin axis

Anemia is a major public health problem worldwide, and iron deficiency is one of its most common causes (166). If iron deficiency is left untreated, microcytic anemia may develop, leading to mental and physical symptoms such as weakness, fatigue, and difficulties with concentration and learning—all of which negatively impact quality of life. While iron deficiency and iron-deficiency anemia can be genetic in some patient groups, they most often occur during periods when the body’s iron requirements increase, such as pregnancy and growth. Furthermore, the most common cause of iron deficiency anemia is an iron-poor diet lacking in animal-derived foods (2).

In the development of iron deficiency, not only nutritional deficiencies and increased requirements but also changes in iron metabolism associated with inflammation play a significant role. Pro-inflammatory cytokines such as IL-6, produced in response to inflammation in the body, cause an increase in plasma hepcidin levels (18). Hepcidin is a hormone synthesized by the liver and is the primary regulator of the body’s iron homeostasis. The primary function of this hormone is to bind to ferroportin—a protein that facilitates the transport of iron from cells to the blood via the cell membrane in enterocytes, hepatocytes, and macrophages—and to trigger the internalization and degradation of ferroportin transporter proteins within intestinal cells. As a result of this process, the number of ferroportins decreases, and the transfer of iron into the blood is reduced or slowed (19).

When plasma iron levels rise, hepcidin production increases, and iron absorption from the intestines and the release of iron stored in the liver into the bloodstream decrease. When plasma iron levels decrease, the opposite mechanism occurs (19). While inflammation-induced hepcidin elevation restricts intestinal iron absorption as a host defense mechanism (detailed in Section 3.1), persistent activation under chronic inflammatory conditions triggers anemia of inflammation. However, in diseases that cause a state of persistent chronic inflammation, hepcidin levels that remain elevated for an extended period can trigger the development of anemia, leading to a condition known as “anemia of inflammation” (AI) (18).

### 3.2 Pathophysiological Iron deficiency in gastrointestinal diseases

In addition to iron deficiency resulting from chronic inflammation, various gastrointestinal and systemic diseases and clinical conditions can lead to the development of iron deficiency and iron-deficiency anemia through different pathophysiological mechanisms. For example, conditions such as celiac disease, inflammatory bowel diseases, *Helicobacter pylori* infection, and aging can play a significant role in altering the body’s iron balance.

#### 3.2.1 Celiac disease

Celiac disease is a genetic, autoimmune disorder that causes atrophy of the intestinal mucosa due to gluten intolerance. Iron-deficiency anemia is a common condition in people with celiac disease, affecting up to 40% of patients. In patients with celiac disease, mucosal atrophy and inflammation in the villi of the duodenum—where most of the iron absorption occurs—reduce iron absorption, leading to iron-deficiency anemia in the long term (20). For the treatment of iron deficiency anemia in patients with celiac disease, intravenous iron supplementation is used during the active phase, while oral iron supplementation is used in patients whose intestinal mucosa has returned to normal following a gluten-free medical diet. In some patients whose intestinal mucosa has healed, it has been observed that iron deficiency anemia may resolve without the need for iron supplementation, thanks to the restoration of normal iron absorption. However, there are also patient groups in whom iron deficiency anemia persists and who are resistant to oral iron supplementation, even after the intestinal mucosa has returned to normal. In these patients, periodic intravenous iron supplementation is required to treat iron deficiency anemia (21).

#### 3.2.2 Inflammatory bowel diseases (IBD—Crohn’s disease and ulcerative colitis)

Crohn’s disease and ulcerative colitis, which are characterized by inflammation in a portion of the gastrointestinal tract, lead to the development of many symptoms, primarily iron-deficiency anemia. Crohn’s disease and ulcerative colitis fall under the category of inflammatory bowel diseases. In IBD patients, blood loss due to mucosal ulcerations, inadequate dietary iron intake, and impaired iron absorption caused by duodenal involvement or surgical resection can trigger iron-deficiency anemia. Additionally, increased levels of pro-inflammatory cytokines such as IL-6 and TNF- α associated with the inflammatory state activate the hepcidin-ferroportin pathway, leading to reduced iron absorption. In these patient groups, the use of high-dose oral iron supplements can lead to increased oxidative stress and exacerbate mucosal damage and inflammation.

In addition, iron that remains unabsorbed in the intestine following high-dose oral iron supplementation causes disruptions in the gut microbiota, leading to the development of dysbiosis characterized by a decrease in the number of beneficial bacteria and an increase in the number of pathogenic bacteria. Clinical studies conducted in patients with IBD have reported that intravenous iron formulations are more effective in treatment compared to oral iron formulations (22). It is of great importance to closely monitor IBD patients for anemia and to treat iron-deficiency anemia that may develop during this period with appropriate medical nutrition therapy and iron supplementation.

### 3.3 Other physiological and chronic conditions: aging and pregnancy

#### 3.3.1 Aging and inflammation

Age can have a distinct effect on cellular and systemic iron levels. It has been observed that as people age, iron accumulation in parenchymal tissues increases due to chronic diseases, genetic mutations, supplement use, and other factors. On the other hand, iron deficiency and anemia are highly prevalent in the geriatric population. Iron deficiency and anemia are common in the elderly population due to factors such as inadequate intake, reduced iron absorption caused by age-related decreases in gastric acid secretion and atrophic gastritis, increased iron loss, and the presence of chronic diseases (23). Furthermore, chronic low-grade inflammation associated with aging (inflammaging) leads to increased hepcidin levels and, in the long term, may contribute to the development of “anemia of chronic disease” and iron accumulation in parenchymal tissues (24). Consequently, as people age, the body’s iron homeostasis begins to deteriorate in ways that can lead to either iron deficiency or iron overload. For this reason, it is important for older adults to adhere to the recommended daily iron intake and undergo regular clinical follow-up to maintain iron levels within the normal range.

#### 3.3.2 Pregnancy

Another physiological condition that alters iron metabolism is pregnancy. Iron-deficiency anemia is a health problem that affects a significant proportion of pregnant women. During pregnancy, the body’s iron requirements increase. The pregnant woman’s iron stores and dietary iron intake may not be sufficient to meet this need, and anemia may develop in the mother (25). In a normal pregnancy, hepcidin levels decrease to meet the increased iron requirement. This enhances iron absorption from the intestines and the release of stored iron into the bloodstream (168). Due to increased erythropoiesis and an expansion of maternal blood volume during pregnancy, the demand for iron rises significantly. When the pregnant woman’s iron stores and dietary iron intake are insufficient to meet this need, iron deficiency and iron-deficiency anemia may develop (25). For this reason, regular clinical follow-up during pregnancy and the use of supplements when necessary are of great importance for maternal and infant health.

## 4 Iron and infection dynamics

### 4.1 Nutritional immunity

The bioavailability of dietary iron not only affects the host’s iron status but can also indirectly influence the iron balance and competition between the host and pathogens. The amount of iron in the intestinal lumen determines not only the composition of the microbiota but also the competition for iron between the host and pathogenic microorganisms. This situation demonstrates that iron metabolism requires strict homeostatic regulation (26, 27).

Systemic iron homeostasis presents a physiological dilemma for the host. When examining infection models related to this, it is clearly evident that the host’s iron levels prior to infection influence the outcome of the disease. For example, in Salmonella infection in mice, diet-induced iron deficiency weakens antimicrobial immunity; a study found that iron supplementation in anemic hosts worsened the course of infection by increasing the pathogen’s iron utilization and disrupting antimicrobial immune responses (28).

Iron is of vital importance to animals and microorganisms due to its redox potential. Iron acts as a cofactor in many biological processes, such as mitochondrial respiration, DNA synthesis, and the citric acid cycle. For bacteria in particular, iron plays a critical role in various processes, including proliferation, biofilm formation, and the expression of different virulence factors. For this reason, the host organism and pathogenic microorganisms are in constant competition for iron within the body (4, 29).

When host organisms encounter pathogens, they aim to reduce the level of potential free iron available to bacteria by decreasing the bioavailability of free iron through the “nutritional immunity” mechanism (Figure 2) (30). As the host organism lowers its iron levels, pathogenic microorganisms are unable to find free iron (29), thereby reducing their virulence and reproductive capacity (31). The mechanism of “nutritional immunity” exhibited by the host during infection was first demonstrated in 1946, when it was observed that the host’s iron levels dropped significantly within 48 h following *Staphylococcus aureus* inoculation (4).

**Figure 2:**
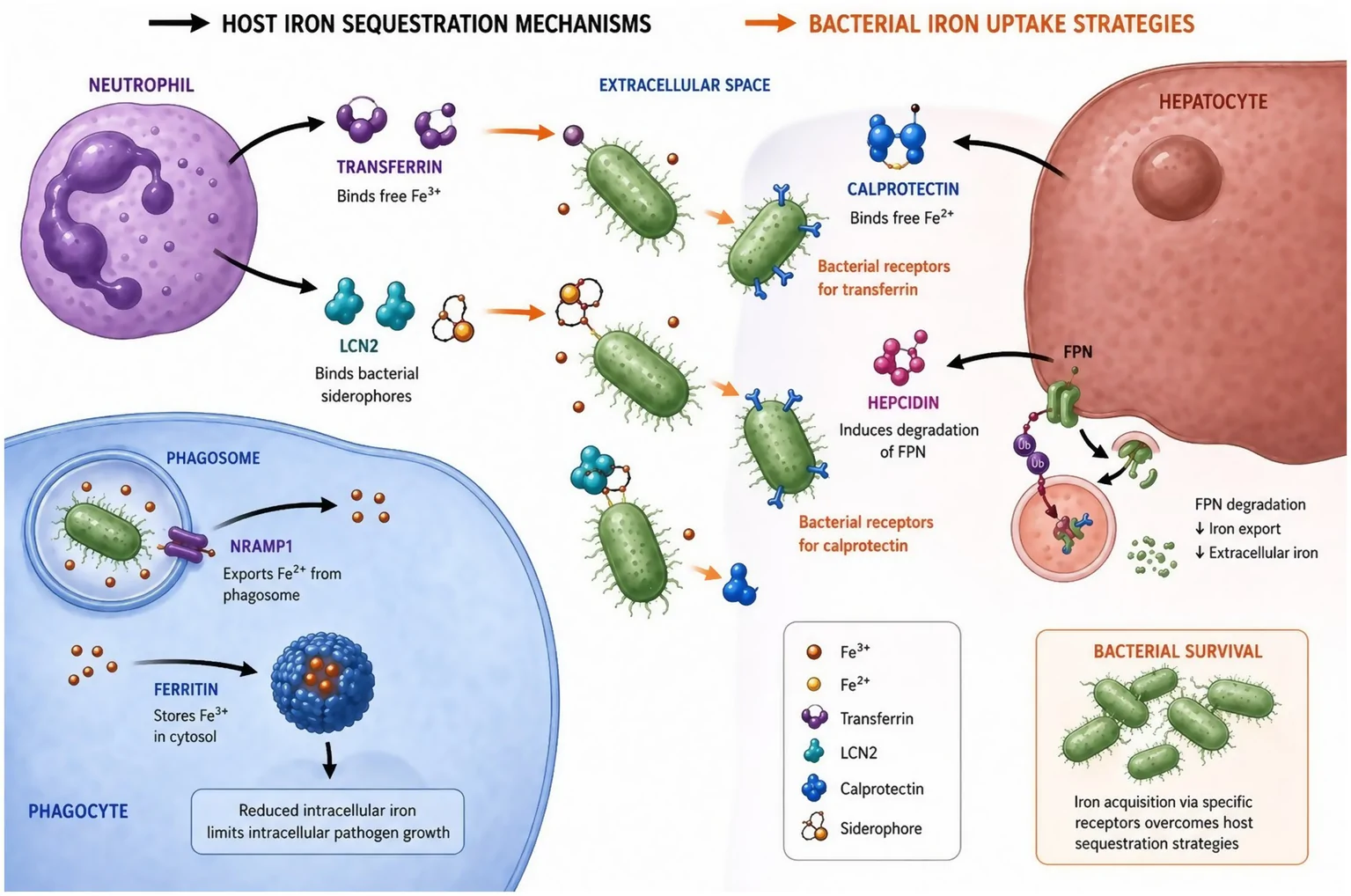
Host nutritional immunity and bacterial iron acquisition strategies. In the host, neutrophils limit bacterial iron bioavailability by binding free Fe^3+^ via transferrin and bacterial siderophores via LCN2 (Lipocalin-2). Hepcidin, secreted by hepatocytes, reduces extracellular iron levels by degrading the ferroportin (FPN) receptor. Calprotectin inhibits pathogen proliferation by binding free Fe^2+^. Within the phagocyte, NRAMP1 transports Fe^2+^ from the phagosome to the cytosol, while ferritin stores Fe^3+^, thereby reducing intracellular iron levels and limiting intracellular pathogen proliferation. In response to these constraints, bacteria develop adaptive mechanisms—such as the production of siderophores and unique surface receptors that bind directly to transferrin and calprotectin—to overcome the host’s “nutritional immunity” mechanisms and ensure their survival.

In healthy individuals, iron levels are regulated through hepcidin-mediated mechanisms. Hepcidin’s role is to establish “nutritional immunity” by limiting the availability of free iron to intracellular and extracellular pathogens. Inflammatory responses trigger the production of interleukin (IL)-6, which in turn increases hepcidin production. Hepcidin reduces gastrointestinal iron absorption by decreasing ferroportin expression and causes iron to remain within macrophages. In addition, the role of unsaturated transferrin is to bind to free iron in the blood and transport it to cells for storage. This synchronized process creates an iron-deficient environment for extracellular pathogens (e.g., *Staphylococcus aureus*) by limiting systemic iron availability (32). However, this mechanism gives rise to an ‘infection paradox’: while systemic iron withdrawal effectively starves extracellular microbes, the resulting intracellular iron sequestration within macrophages can paradoxically enhance the virulence and persistence of intracellular pathogens (e.g., Chlamydia, Legionella, *Salmonella enterica*, and Mycobacteria spp.) and impair adaptive immune cell proliferation (33–35). As an example of this phenomenon, intracellular iron levels resulting from cell lysis provide host defense against the blood stages of *Plasmodium* infection while simultaneously increasing susceptibility to *Salmonella* (36).

Because the evidence presented on this topic in the past has been inconclusive, there remains a need to investigate the relationship between IV iron use and infection, as well as its impact on morbidity and mortality rates. A recent study found that newer IV iron preparations result in lower free iron concentrations and reduce the risk of infection (37) It was analyzed that the form of iron administered resulted in varying infection risks among different patient groups in the study (37). IV iron therapy can increase levels of iron not bound to transferrin in the circulation (165). It has been suggested that frequent administration of high-dose IV iron sucrose reduces the intracellular killing ability of neutrophils and leads to decreased survival rates in dialysis patients, in addition to increased hospitalization rates (38).

Recent observational studies, clinical trials, and systematic reviews have suggested that there is no association between IV iron infusion and the risk of infection in various patient groups, such as kidney transplant recipients, patients undergoing femur fracture surgery, and hemodialysis patients (39–41). While IV iron worsens sepsis outcomes in mouse (murine) models (42, 43), no definitive association has been established between high-dose IV iron and increased mortality in humans; underlying comorbidities have been considered (44–46).

Examples of groups at risk for developing an infection while undergoing iron therapy include patients with chronic kidney disease, dialysis patients, those receiving long-term or high-dose iron therapy, those with high serum ferritin levels (>500–1,000 ng/mL), and those using erythropoietin in conjunction with iron therapy. Advanced age (>60 years), catheter use, and a history of infection are also factors that contribute to this risk (37, 47–51) In addition, it is known that geographic conditions (e.g., regions where malaria is endemic) and conditions such as inflammatory bowel disease can have a more pronounced impact on outcomes (52). More recent IV iron formulations, with their low labile iron content and improved safety profiles, have not been shown to increase the risk of infection. Administration at the lowest effective dose is considered a safer option regarding the risk of infection. Formulations that provide lower levels of free iron, such as ferric carboxymaltose and ferumoxytol, should be preferred, and the patient’s infection status should be assessed before initiating iron therapy (53).

Experimental studies conducted *in vivo* have shown that plasma iron levels directly affect immune system cells and their function (54). In cases of infection and inflammation, pro-inflammatory signals—primarily interleukin-6—increase hepcidin production and, by acting on ferroportin, reduce iron release into the blood. In addition, cytokines, microbial products, and acute-phase proteins increase iron uptake and storage in macrophages through various hepcidin-independent pathways (55–57). Hypoferremia develops as a consequence of the inhibition of ferroportin-mediated iron release into the bloodstream and its storage within macrophages. Hypoferremia is a condition characterized by low plasma iron levels, in which the body’s iron is largely stored within cellular ferritin and heme pools (19, 58). Hepcidin-induced hypoferremia selectively limits granulopoiesis by increasing the production of transferrin receptor 1 (TfR1) and reducing the iron-dependent proliferation and differentiation capacity of neutrophil progenitors. In contrast, monocytes and macrophages are unaffected by this condition and continue to produce cytokines and exhibit antimicrobial activity (54).

*In vivo* models of diet-induced iron deficiency have shown that while this condition does not affect antigen recognition, it is associated with impaired CD8 + T-cell proliferation and reduced cytokine production in the context of viral infection and vaccination (59–61). Similarly, genetic defects in cell-specific iron uptake—such as hypomorphic mutations in the transferrin receptor gene (TFRC), which are associated with impaired lymphocyte development and combined immunodeficiency—emphasize that iron-dependent signaling is indispensable for immune system functions, even when systemic iron parameters remain within normal ranges (62, 63). Iron has also been found to play a role in B-cell proliferation and antibody responses, in addition to T lymphocytes (64). Furthermore, more recent studies show that iron uptake via the transferrin receptor contributes to the metabolic competence and effector function of innate lymphoid cells, such as natural killer (NK) cells and ILC3 (65, 66). All of these observations have highlighted that myeloid and lymphoid cells have different regulatory mechanisms for iron utilization. While myeloid cells primarily use iron as part of antimicrobial defense and inflammatory control, in lymphoid cells, iron influences proliferation, metabolic activation, and effector differentiation. Particularly in situations where systemic iron is limited, immune system activities mediated by these cells may be impaired, leaving the organism more vulnerable (67).

### 4.2 Pathogen-specific mechanisms of Iron acquisition and nutritional immunity

To fully align with the complex clinical realities of host-pathogen interactions, the molecular strategies employed by specific infectious agents across distinct anatomical niches must be detailed.

#### 4.2.1 Gastric pathogenesis: *Helicobacter pylori*

*Helicobacter pylori* infection, particularly in the setting of chronic atrophic gastritis, severely compromises non-heme iron absorption by causing hypochlorhydria and decreasing gastric mucosal ascorbic acid levels, which together inhibit the reduction of dietary ${\mathrm{Fe}}^{3+}$ to absorbable ${\mathrm{Fe}}^{2+}\;$ (68). Beyond localized absorption defects, *H. pylori* directly competes with the host for luminal iron, serving as a primary driver of refractory iron deficiency anemia (IDA) (69, 70, 161). Although lacking classical siderophores, *H. pylori* expresses an array of iron-transport systems regulated by the Ferric Uptake Regulator (Fur), including the high-affinity ferrous iron transporter (FeoB), the ferric dicitrate receptor (FecA), and non-heme iron-containing ferritin (Pfr) (71). Under iron-restricted conditions, outer membrane protein (OMP) genes such as *fecA* and *frpB* are upregulated (72), whereas iron-replete conditions restrict expression to specific OMP variants (71, 73).

Furthermore, synergistic actions of virulence factors Cytotoxin-associated gene A (CagA) and Vacuolating cytotoxin A (VacA) mislocalize transferrin and its receptor from the basolateral membrane to the apical surface of epithelial cells, facilitating bacterial acquisition of holo-transferrin iron (74). *H. pylori* also manipulates systemic iron homeostasis; its lipid A component triggers IL-6 and IL-1β production, inducing hepatic hepcidin synthesis, which sequesters iron in macrophages and reduces systemic iron availability (75, 76). Genetic variations, such as the *thr70* variant of Neutrophil Activating Protein A (NapA) (77) and heightened expression of Sialic Acid-Binding Adhesin (SabA) alongside VacA and TNF- α upregulation, further enhance bacterial iron uptake capacity and exacerbate clinical IDA (72, 78).

#### 4.2.2 Enteric pathogens and siderophore competition: *Salmonella* and *Escherichia coli*

In the intestinal tract, enteric pathogens engage in metabolic iron competition via high-affinity catecholate siderophores, predominantly enterobactin (79, 80). Enterobactin is synthesized under iron-deprived conditions, exported via EntS and TolC (81), and re-imported via specific outer membrane receptors (FepA and IroN) (82). By scavenging labile iron, enterobactin deprives host immune cells (macrophages and T cells) of essential iron, blunting their antimicrobial responses (83, 84).

During *Salmonella enterica* or *Klebsiella pneumoniae* infections, iron acquisition directly correlates with colonization capacity and systemic virulence (85, 86). In *Salmonella*, Fur coordinates motility, invasion, and intramacrophage survival (87). Although the host secretes Lipocalin-2 (LCN2) to sequester enterobactin (88), *Salmonella* counteracts host nutritional immunity by producing salmochelin—a C-glucosylated form of enterobactin whose sterically hindered structure prevents binding to the LCN2 calyx (89, 90, 162). Intramacrophage *Salmonella* further modulates iron traffic by inducing ferroportin downregulation and boosting hepatic hepcidin via TLR4-dependent mechanisms (91, 92).

#### 4.2.3 Endemic contexts: Iron homeostasis and malaria

The clinical intersection of host iron status and infection is critically illustrated in *Plasmodium falciparum* malaria. Epidemiological evidence from endemic regions indicates that iron deficiency exerts a protective effect against severe malaria and parasitemia in children and pregnant women (93, 94). Rather than solely limiting nutrient availability, recent clinical data reveal a biomechanical mechanism: altered erythrocyte parameters (e.g., reduced mean corpuscular volume, MCV) during iron deficiency impair parasite invasion and intravascular propagation, demonstrating how host iron status dictates disease outcomes through structural and bioenergetic constraints (95).

### 4.3 Dual nature of Iron dynamics: adaptive Defense vs. pathological changes

To clarify the mechanistic narrative during infection, shifts in host iron homeostasis must be categorized into two functionally distinct pathways: (i) *reactive and adaptive responses* designed for nutritional immunity, and (ii) *pathological changes* that drive oxidative stress, tissue injury, and cell death.

#### 4.3.1 Reactive and adaptive responses: intracellular iron trafficking and nutritional immunity

Building upon the cellular mechanisms of nutritional immunity outlined in Section 3.1, intracellular pathogens within phagosomes are subjected to targeted iron deprivation primarily mediated by Nramp1, which translocates divalent cations out of the phagolysosomal lumen into the cytosol to restrict microbial iron availability, though adapted pathogens like *Salmonella* and *Leishmania* have evolved distinct counter-strategies to subvert this vacuolar iron depletion (96–98). In response, intracellular adapted pathogens such as *Salmonella enterica* serovar Typhimurium and *Leishmania major* have evolved counter-strategies to destabilize Nramp1 or access alternative iron pools (99–101) Concurrently, transferrin receptor 1 (TfR1)-mediated iron ( ${\mathrm{Fe}}^{2+}$ ) uptake undergoes endosomal acidification and cytosolic delivery via divalent metal transporter 1 (DMT1) (102).

DMT1 maintains the balance between vesicular iron retention and cytosolic availability, a process finely regulated by inflammatory cues (56). While non-infectious inflammatory conditions (e.g., neurodegenerative diseases and osteoarthritis) exhibit pro-inflammatory cytokine-induced DMT1 upregulation leading to cytotoxic iron overload (103, 104), macrophage-specific deletion of DMT1 during *Salmonella* infection dampens Lipocalin-2 (LCN2) induction, providing labile iron to the pathogen and enhancing bacterial replication (105). Furthermore, loss of phagosomal membrane DMT1 impairs iron export from pathogen-containing vacuoles, altering downstream inflammatory signaling (106). Lysosomal iron handling actively feeds back into immune signaling. Iron released from lysosomal storage blunts the production of pro-inflammatory cytokines (TNF- α , IL-1β, IL-6) in monocytes and macrophages (107, 108). Mechanistically, lysosomal ${\mathrm{Fe}}^{2+}$ triggers reactive oxygen species (ROS) production, activating the lysosomal cation channel TRPML1 to induce cytosolic ${\mathrm{Fe}}^{2+}$ release. Elevated cytosolic iron activates prolyl hydroxylases, suppresses NF- κ B activity, and downregulates IL-1β transcription (109). Crucially, intracellular iron intersects with metabolic checkpoints, particularly the mechanistic target of rapamycin complex 1 (mTORC1) (110, 111). Active mTORC1 promotes a Warburg-like metabolic state characterized by elevated glycolysis and suppressed tricarboxylic acid (TCA) cycle activity, fueling the energetic demands of cytokine synthesis (112). Iron supplementation partially restores aerobic metabolism by activating TCA cycle enzymes; however, under mTOR inhibition, iron supplementation exacerbates bacterial persistence. Iron availability also modulates phagolysosomal maturation; in *Mycobacterium avium* infection, intracellular iron overload blocks phagolysosomal maturation, whereas iron chelation restores phagosome fusion and bacterial clearance (113). Ultimately, because mitochondria serve as the primary site of iron utilization for TCA cycle and oxidative phosphorylation enzymes, intracellular iron fluxes dictate bioenergetic capacity, immune cell longevity, and functional host responses (114–116).

#### 4.3.2 Pathological changes: uncontrolled Ferritinophagy, organ damage, and Sepsis

The intracellular labile iron pool (LIP) represents a transient, chelatable pool of redox-active iron (Fe^2+^) within the cytoplasm and organelle compartments, critical for cellular iron homeostasis and toxicity (117). While controlled ferritin turnover sequesters iron away from pathogens, pathological activation of ferritinophagy disrupts cellular buffering capacity and triggers organ injury. Under acute inflammatory conditions or specific viral challenges (e.g., Pseudorabies virus), co-activation of iron uptake pathways and nuclear receptor coactivator 4 (NCOA4)-mediated ferritinophagy expands the labile iron pool (LIP), precipitating cytotoxic cascades that pathogens exploit for replication (118–120). Similarly, intracellular bacterial effectors (e.g., *Ehrlichia chaffeensis*) selectively induce ferritinophagy to liberate iron for intracellular proliferation (121).

In severe clinical contexts such as sepsis-induced cardiac injury, lipopolysaccharide (LPS) exposure drives massive NCOA4-dependent ferritin degradation, excessive cytosolic ${\mathrm{Fe}}^{2+}$ release, mitochondrial dysfunction, lipid peroxidation, and tissue destruction (11). In cardiomyocytes, NCOA4-mediated ferritinophagy promotes mitochondrial iron overload via the mitochondrial iron transporter sideroflexin-1 (SFXN1), elevating mitochondrial ROS and inducing pathological cardiac remodeling (120, 122). Genetic or pharmacological suppression of ferritinophagy restores cellular iron buffering, preserves mitochondrial integrity, and mitigates systemic tissue injury, directly linking dysregulated lysosomal iron release to organ failure (11, 122).

### 4.4 Molecular Cascade of Iron-induced cell death: ferroptosis

Unrestrained cellular iron accumulation culminates in ferroptosis—a distinct form of regulated, non-apoptotic cell death characterized by iron-dependent lipid peroxidation and membrane destruction (123, 124). Ferroptosis reflects a fundamental breakdown of redox homeostasis where pro-oxidant factors (catalytically active ${\mathrm{Fe}}^{2+}$ and ROS) overwhelm cellular antioxidant defenses (125, 126).

Excessive iron uptake via transferrin or NCOA4-mediated ferritinophagy fuels the Fenton reaction, generating hydroxyl radicals ( ${\mathrm{OH}}^{�}$ ) that oxidize polyunsaturated fatty acids (PUFAs) within membrane phospholipids (127, 128). The primary defense against ferroptotik damage is driven by the glutathione (GSH)/glutathione peroxidase 4 (GPX4) axis, which converts toxic lipid hydroperoxides into non-toxic lipid alcohols. Parallel surveillance systems include Ferroptosis Suppressor Protein 1 (FSP1), which reduces ubiquinone/ α -tocopherol to halt lipid radical propagation, and the dihydroorotate dehydrogenase (DHODH)/mitochondrial coenzyme Q10 axis, which protects mitochondrial inner membranes from ferroptotik disruption (129, 130). Additional protective mechanisms involve squalene and di/tetrahydrobiopterin ( ${\mathrm{BH}}_{2}/{\mathrm{BH}}_{4}$ ) accumulation. When these antioxidant networks are overwhelmed, lipid peroxides break down into toxic aldehydes—such as malondialdehyde (MDA) and 4-hydroxynonenal (4-HNE)—precipitating irreversible membrane lysis and cell death (125).

## 5 Clinical iron management: from pathophysiology to therapeutic interventions

### 5.1 Pathophysiological mechanisms and anemia of inflammation (AI)

#### 5.1.1 Intestinal iron dynamics, inflammation-induced hypoferremia and mucosal damage

Iron homeostasis is paramount for preserving intestinal mucosal integrity. As detailed in Section 3.1, systemic hypoferremia serves as an adaptive nutritional immunity response driven by IL-6–induced hepcidin elevation. However, when this mechanism persists chronically, prolonged intracellular iron retention within enterocytes and macrophages promotes local redox-active iron accumulation (18). When cellular antioxidant defenses are overwhelmed, this cascade triggers ferroptosis—a regulated cell death mechanism characterized by iron-dependent lipid peroxidation (125). Ferroptotic enterocyte death compromises the intestinal epithelial barrier, facilitating pathogen proliferation and promoting pro-inflammatory cytokine secretion, thereby establishing a self-amplifying vicious cycle of inflammation and oxidative stress. This mechanism is particularly clinically relevant in Inflammatory Bowel Disease (IBD). During active inflammation, neutrophil and macrophage infiltration amplifies ROS production, which, combined with chronic micro-bleeding and local iron deposition, aggravates mucosal redox imbalance. Consequently, chronic iron retention shifts inflammation-induced hypoferremia from an adaptive defense into a pathological driver of chronic mucosal injury and tissue degradation (125).

#### 5.1.2 The clinical dilemma: managing anemia of inflammation (AI)

Anemia of Inflammation (AI), commonly secondary to chronic infections, autoimmune disorders, or malignancy, stems from cytokine- and hepcidin-mediated iron sequestration within reticuloendothelial macrophages and impaired intestinal absorption, leading to iron-restricted erythropoiesis characterized by hypoferremia and hyperferritinemia (131, 159, 160). Because AI evolved as a protective nutritional immunity strategy to starve circulating pathogens (132), therapeutic management should not focus solely on normalizing hemoglobin levels without evaluating the underlying inflammatory status and infection risk.

The primary therapeutic objective in AI must be controlling the underlying primary inflammatory disease. Decisions regarding iron replacement therapy—and its administration route—should be strictly individualized using biomarkers such as serum hepcidin, systemic iron indices, and inflammatory activity (131). Uncritical iron administration during active infection or in endemic settings poses severe risks. As mechanically illustrated in Section 3.2.3, host iron status directly modulates pathogen susceptibility; accordingly, clinical trials in infants in malaria-endemic regions demonstrated that indiscriminate iron supplementation significantly increased morbidity and mortality from malaria and enteric infections. Thus, managing AI requires a tailored approach that carefully distinguishes functional iron sequestration driven by inflammation from true absolute iron deficiency before initiating supplementation.

### 5.2 Clinical biomarkers for iron replacement therapy

The clinical presentation of iron deficiency and iron deficiency anemia, as well as the response to treatment, can vary significantly depending on the underlying cause and severity, as well as age, physiological status, inflammation, and comorbidities. Therefore, rather than a standardized approach to iron replacement therapy, the combined evaluation of biomarkers and clinical signs reflecting the patient’s iron status and response to treatment is of crucial importance in determining the appropriate replacement strategy (133, 134).

Evaluating a single biomarker is insufficient for individualizing iron replacement therapy; a comprehensive approach assessing iron status, inflammatory burden, underlying physiological conditions, and treatment response together should serve as the baseline framework. Although ferritin is evaluated alongside hemoglobin in the diagnosis of iron deficiency and iron deficiency anemia and is one of the primary indicators of body iron stores, as an acute-phase reactant, it can increase in the presence of inflammation, thereby masking true iron deficiency. For this reason, interpreting ferritin in combination with transferrin saturation (TSAT) and inflammatory markers becomes particularly critical in distinguishing absolute iron deficiency from inflammation-induced functional iron restriction. Reticulocyte hemoglobin content (CHr/Ret-He), reflecting bioavailable iron for erythropoiesis over the preceding 3–4 days, is known to contribute to the evaluation of iron-restricted erythropoiesis and early treatment response.

Hepcidin, another important biomarker, is gaining increasing significance in assessing diagnostic status and therapeutic efficacy. Suppressed hepcidin levels indicate an increased physiological demand for iron and a higher probability of response to oral iron therapy, whereas inflammation-driven elevated hepcidin levels suggest functional iron restriction due to impaired intestinal iron absorption and limited ferroportin-mediated iron export. Nevertheless, it must be borne in mind that because hepcidin measurement is not yet sufficiently standardized for routine clinical practice, biomarker-driven clinical diagnosis and treatment decisions should not rely solely on hepcidin levels (133).

The biomarker profile also holds decisive clinical value in selecting the route of administration (oral vs. intravenous). Iolascon et al. (133) state that the choice between oral or intravenous iron should not be made based on hemoglobin levels alone, but according to variables such as underlying etiology, patient age, symptoms, comorbidities, tolerance to oral therapy, and response to prior treatments. While oral iron replacement remains the first-line therapeutic approach in most cases of iron deficiency, intravenous iron should be evaluated in the presence of inadequate response to oral therapy, gastrointestinal intolerance, or malabsorption (133). In a multicenter randomized controlled trial conducted by Koppelman et al. (134) in patients with active inflammatory bowel disease, the potential contribution of this biomarker-based strategy to route selection was demonstrated. The study showed that higher baseline hepcidin levels were associated with a reduced likelihood of response, specifically to ferrous fumarate, whereas lower baseline hepcidin levels rendered a response to oral therapy more probable, suggesting that hepcidin could serve as a biomarker aiding the selection between oral and intravenous replacement regimens. However, the same study noted that due to the lack of standardization in hepcidin assays, more accessible parameters based on ferritin and transferrin could also be used to evaluate potential treatment response (134).

The interpretation of biomarkers and the choice of replacement strategy must not be considered in isolation from age, pregnancy status, degree of inflammation, and comorbidities. Specific clinical examples of this include the variation of diagnostic thresholds for ferritin and hemoglobin based on age and pregnancy, the challenge of interpreting ferritin in older adults due to chronic inflammation and comorbidities—making complementary parameters like TSAT indispensable—or the increased iron demand during pregnancy altering both diagnostic and therapeutic management (133). For instance, while oral iron replacement is the primary therapeutic option during pregnancy, current obstetric consensus guidelines recommend evaluating intravenous iron replacement in cases of intolerance/inadequate response to oral therapy or when rapid replenishment is required in advanced gestational weeks (135).

In conclusion, identical ferritin or TSAT values do not carry the same clinical implication in a young healthy individual, a patient with active inflammatory disease, an older multimorbid individual, or a pregnant woman. In individualized iron replacement therapy, the biomarker profile must be evaluated holistically alongside inflammatory status, physiological stage, comorbidities, gastrointestinal absorptive capacity, and treatment response, and the choice between oral and intravenous routes should be guided by this integrated framework.

### 5.3 Route selection and formulation profiles: oral vs. intravenous iron

#### 5.3.1 Conventional iron supplements

Iron supplements can be used orally or intravenously to treat various diseases in humans (136, 137). Oral iron supplements are frequently preferred because they are easier to use, are mostly available without a prescription, and are less expensive. However, treatment of iron deficiency with oral iron supplements requires relatively high doses—such as 50–200 mg of elemental iron per day—for 3–12 weeks. Only about 10–20% of this amount is absorbed, and the unabsorbed portion leads to excess iron accumulation in the digestive system and associated gastrointestinal side effects. Furthermore, the presence of this unabsorbed excess iron in the digestive system can disrupt the composition and diversity of the microbiota, shifting the microbiota balance toward pathogenic bacteria (138, 139).

Today, there are numerous commercially available oral iron supplements in various forms (Table 1). These fall into two main classes: iron (Fe^2+^) salts and iron (Fe^3+^) complexes. Other types include carbonyl iron and heme iron polypeptide (163).

**Table 1:** Comparison of oral iron supplements in terms of elemental iron content.

Ferrous salts	Amount of elemental iron, * mg per tablet	Approximate cost, ** $ per tablet	Advantages	Disadvantages
Ferrous sulfate	65	0.07	Low cost, high efficacy, wide availability	Poor tolerability
Ferrous fumarate	100	0.26		
Ferrous gluconate	35	0.10		
Ferrous bisglycinate	50	0.15		
Ferrous ascorbate	65	0.45		

Ferric complexes	Amount of elemental iron, * mg per tablet	Approximate cost, ** $ per tablet	Advantages	Disadvantages
Ferric polysaccharide (Ferrex™ 150)	150	0.08	Possibly fair tolerability	Relatively low efficacy
Ferric polymaltose (Maltofer®)	100	0.90		
Ferric polydextrose (Feramax® 150)	150	0.85		
Iron protein succinylate (Ferretts®)	40***	3.40***	High efficacy, fair tolerability	High cost
Ferric citrate (Auryxia®)	210	7.18	Efficacy in CKD patients	
Ferric maltol (ACCRUFeR®)	30	8.58	Efficacy and tolerability in IBD patients	High cost, risk of IBD flare
Sucrosomial® iron (SiderAL®)****	30	2.00		High cost, no iron stores rebuild
Liposomal iron	30	0.83		Scarce data, no iron stores rebuild

Others	Amount of elemental iron, * mg per tablet	Approximate cost, ** $ per tablet	Advantages	Disadvantages
Carbonyl iron	18	0.33	Low cost, wide availability	Scarce data, no iron stores rebuild
Heme iron polypeptide (Proferrin-ES®)	12	0.60	Efficacy in CKD patients	

*Amount of elemental iron may vary depending on the product formulation.**Approximate market cost in USD per tablet.***Specific to iron protein succinylate formulation. **** Sucrosomial technology formulation.

While the most commonly used oral iron supplements are iron salts containing sulfate, fumarate, or gluconate, other forms such as iron glycine sulfate, bisglycinate, ascorbate, carbonate, tartrate, iodide, chloride, sodium citrate, aspartate, or succinate are also available (140). According to the guidelines of the British Society of Gastroenterology, iron sulfate, fumarate, or gluconate are recommended as first-line treatment for iron deficiency anemia (IDA) in adults (141). Although iron (Fe^2+^) salts are available in both tablet and syrup forms, maintaining Fe^2+^ in its reduced state in liquid formulations is challenging because dissolved Fe^2+^ readily oxidizes to the less soluble Fe^3+^. This problem can be resolved by adding excipients such as sodium bisulfite (140). Additionally, the liquid form may cause effects such as tooth staining and a metallic taste in the mouth when used long-term or if not properly diluted before ingestion.

Iron (Fe^2+^) salts designed with enteric coating and slow-release formulations are also intended to minimize unwanted gastrointestinal effects while maintaining iron absorption (140). However, in some studies, these slow-release iron salt supplements have demonstrated low efficacy, most likely due to the delayed release of the encapsulated Fe^2+^ past the duodenum and upper jejunum, where iron absorption is optimal. This drawback has raised significant concerns regarding their efficacy (142–144), and their use has not been recommended (141).

Ferrous sulfate, considered the gold standard in oral iron supplementation, is the most frequently prescribed and most cost-effective iron formulation available in many countries. The expected effect of iron salts, including iron sulfate, is a rapid increase in blood iron levels and an effective increase in iron stores alongside hemoglobin production (145, 146). A systematic review and meta-analysis incorporating 43 randomized controlled trials involving a total of 6,831 adults investigated the tolerability and side effects of iron sulfate compared to placebo or intravenous iron (146). It was observed that the use of iron sulfate significantly increased the likelihood of gastrointestinal side effects compared to placebo and intravenous iron. Although this increase was not found to be related to the dose of iron sulfate, no evidence was found to suggest that sustained-release formulations provide better tolerance during treatment (146).

Traditional iron salts (iron fumarate, iron sulfate, iron gluconate) cause various side effects, including dyspepsia, vomiting, nausea, diarrhea, and stomach discomfort (147). In addition, dietary factors, the concurrent use of medications such as proton pump inhibitors or antacids, and chronic inflammatory conditions—which further limit iron absorption via hepcidin elevation—can reduce the gastrointestinal absorption and bioavailability of standard oral iron salts (148).

Furthermore, various foods containing phytates, polyphenols, calcium, and tannins may limit treatment success and delay the achievement of the intended hematological profile by reducing the gastrointestinal absorption of iron preparations through the oxidation of iron to the ferric (Fe^3+^) form (147, 148).

The absorption of non-heme iron from small iron salts or complexes found in supplements such as iron sulfate and iron gluconate is mediated by DMT1 (Divalent Metal Transporter 1). It is a carrier of divalent metal ions (such as Fe^2+^ and Mn^2+^) that has been highly conserved throughout the evolutionary process. In addition to these, it is capable of recognizing toxic ions such as Cd^2+^ and Pd^2+^ and transports all of them from the intestinal lumen into intestinal cells (149). For the Fe^3+^ ion in non-heme iron preparations (such as salts or chelators like EDTA or citrate) to enter intestinal cells, it must first be released from the complex to which it belongs. The released Fe^3+^ is reduced by binding to a reductase enzyme such as dcytb (150) and is released in the form of hydrated Fe^2+^.

The primary treatment approach for iron deficiency anemia is oral iron supplementation. In clinical practice, non-heme iron supplements are generally preferred. The most commonly used oral preparations in clinical practice are iron sulfate, iron fumarate, and iron succinate supplements (151). However, these supplements can cause gastrointestinal side effects such as dyspepsia and constipation, particularly when used at high doses (146). In addition, high-dose iron supplementation may compete with micronutrients such as zinc for absorption, thereby reducing their absorption and increasing the risk of micronutrient deficiency in the long term (10). Therefore, when using iron supplements, it is of great importance to accurately identify the pathophysiological mechanism underlying iron deficiency and to implement appropriate treatment strategies.

#### 5.3.2 Pharmacokinetic, clinical, and safety profiles: oral vs. intravenous iron

Directing therapeutic management in anemia of inflammation requires a precise balance between oral and parenteral modalities, as they exhibit fundamentally distinct mechanisms, bioavailability profiles, and safety considerations. Oral iron relies heavily on active intestinal transport via DMT1 and efflux via ferroportin. In inflammatory states, elevated serum hepcidin blunts oral bioavailability, trapping unabsorbed iron within the intestinal lumen (152). Conversely, intravenous (IV) iron carbohydrate complexes (e.g., ferric carboxymaltose, iron isomaltoside) bypass enteral absorption barriers entirely. IV formulations are endocytosed by reticuloendothelial macrophages, where iron is gradually released and loaded onto transferrin, making it rapidly available for erythropoiesis regardless of systemic hepcidin levels (153).

Oral iron remains first-line for mild-to-moderate absolute iron deficiency without ongoing systemic inflammation. However, IV iron is explicitly indicated in cases of oral intolerance, severe mucosal inflammation (e.g., active Inflammatory Bowel Disease), significant ongoing blood loss, or when high hepcidin levels induce absolute refractoriness to enteral supplementation (154, 155). While oral iron carries a primary risk of localized mucosal damage, lipid peroxidation, and dysbiosis, IV iron bypasses the gut lumen, eliminating local gastrointestinal toxicity (156). However, high-dose IV administration carries a unique risk of transiently exceeding transferrin saturation, leading to the formation of Non-Transferrin-Bound Iron (NTBI) (153). Unlike the intracellular LIP, non-transferrin-bound iron (NTBI) specifically refers to the circulating, non-transferrin-bound iron species in the extracellular plasma that appear when transferrin saturation is exceeded. Free NTBI acts as a potent pro-oxidant and can serve as an accessible iron source for circulating bacterial pathogens. Consequently, while IV iron is highly effective in bypassing enterocyte blockade, its administration must be carefully timed and monitored during active systemic infections (157).

### 5.4 Adverse effects, mucosal damage, and luminal dysbiosis

#### 5.4.1 Reactive oxygen species and mucosal damage

As a result of high-dose iron supplementation or insufficient iron absorption, iron remaining in the intestine without being absorbed can lead to the formation of reactive oxygen species (ROS) through Fenton reactions and cause oxidative stress, which may contribute to the development of intestinal damage, inflammatory bowel diseases, and intestinal barrier dysfunction. In addition, it has been reported that oxidative stress originating in the gut may be associated with various chronic diseases in the long term. Numerous clinical studies indicate that iron remaining unabsorbed in the duodenum may disrupt the gut microbiota by reducing beneficial microorganisms and promoting pathogenic ones, thereby leading to the development of dysbiosis (6). For this reason, it is critically important to properly understand the iron supplementation–iron absorption–microbiota axis and to plan treatment accordingly.

#### 5.4.2 Iron concentration in intestinal lumen and dysbiosis

The host’s iron status and the amount of iron reaching the intestinal lumen via diet or iron supplementation can influence the composition and function of the gut microbiota. Since iron is an essential growth factor for many bacteria, changes in the amount of iron in the intestinal lumen can directly affect the balance between the host and the gut microbiota. Although iron is an important cofactor in many biological processes, it can be toxic at high doses due to its redox potential. For this reason, iron homeostasis is tightly regulated in the body. It is estimated that the concentration of free iron in the human body is 8 times lower than the concentration required for microbial growth. However, since the vast majority of dietary iron is not absorbed in the colon, iron concentrations there may be higher than in the systemic circulation. Clinical studies indicate that iron concentrations in the colon are sufficient to support microbial growth (158). However, in healthy individuals, intestinal homeostasis, microbiota balance, and host defense/immune mechanisms prevent the uncontrolled proliferation of pathogens. High-dose iron supplementation or the accumulation of iron remaining in the intestinal lumen after absorption can disrupt this balance, increasing the amount of bioavailable iron and contributing to pathogen proliferation; additionally, increased oxidative stress and mucosal damage caused by iron’s redox potential can trigger inflammation, leading to the development of dysbiosis. Similarly, in clinical conditions that reduce iron absorption—such as aging, celiac disease, and inflammatory bowel diseases—iron remaining in the colon without being absorbed can lead to changes in the microbiota composition and the development of dysbiosis. The potential effects of high-dose iron supplementation on iron concentration in the intestinal lumen, the microbiota, and inflammation are schematically illustrated in Figure 3.

**Figure 3:**
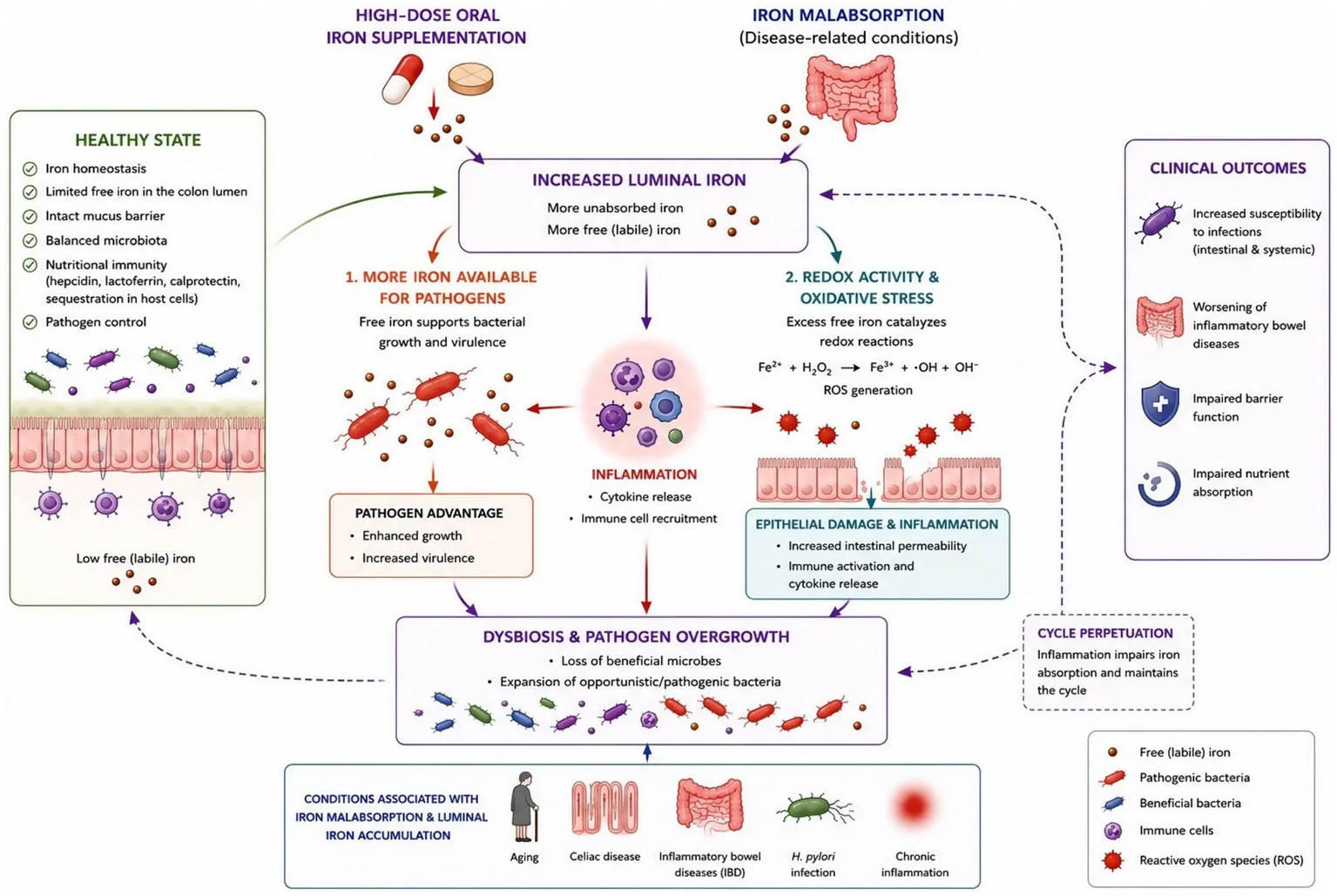
Proposed effects of increased intestinal iron availability on host-microbiota interactions and antimicrobial efficacy. In healthy individuals, low levels of free (labile) iron, a healthy mucosal barrier, and a balanced microbiota composition support the “nutritional immunity” mechanism. High-dose oral iron supplementation or disease-related iron malabsorption (aging, celiac disease, IBD, *Helicobacter pylori* infection, chronic inflammation) increases luminal free iron levels. This increase leads to pathogen growth through two parallel pathways: (1) increased luminal iron supports the growth and virulence of pathogenic bacteria; (2) high levels of free iron trigger the production of reactive oxygen species (ROS) via the Fenton reaction (Fe^2+^ + H₂O₂ → Fe^3+^ + ·OH + OH^−^), leading to epithelial damage and inflammation. Both pathways trigger dysbiosis and pathogen overgrowth. The resulting inflammation further impairs iron absorption via the hepcidin-ferroportin axis, perpetuating the vicious cycle. Clinical consequences include increased susceptibility to infections, exacerbation of inflammatory bowel diseases, intestinal barrier dysfunction, and malabsorption of nutrients.

In healthy individuals, low levels of free (labile) iron, a healthy mucosal barrier, and a balanced microbiota composition support the “nutritional immunity” mechanism. High-dose oral iron supplementation or disease-related iron malabsorption (aging, celiac disease, IBD, *H. pylori* infection, chronic inflammation) increases luminal free iron levels. This increase leads to pathogen growth through two parallel pathways: (1) increased luminal iron supports the growth and virulence of pathogenic bacteria; (2) high levels of free iron trigger the production of reactive oxygen species (ROS) via the Fenton reaction (Fe^2+^ + H₂O₂ → Fe^3+^ + ·OH + OH^−^), leading to epithelial damage and inflammation. Both pathways trigger dysbiosis and pathogen overgrowth. The resulting inflammation further impairs iron absorption via the hepcidin-ferroportin axis, perpetuating the vicious cycle. Clinical consequences include increased susceptibility to infections, exacerbation of inflammatory bowel diseases, intestinal barrier dysfunction, and malabsorption of nutrients.

## 6 Conclusion

Iron homeostasis represents a finely tuned physiological continuum that stands at the critical intersection of host metabolic vitality, nutritional biochemistry, and antimicrobial defense networks. As comprehensively delineated in this review, the processes governing intestinal iron absorption, physical systemic recycling via the macrophage-erythroid axis, and intracellular trafficking are tightly orchestrated under steady-state conditions to prevent both the toxic perils of tissue iron overload and the systemic impairments associated with severe iron deficiency. The molecular discovery of the hepcidin-ferroportin axis has profoundly reshaped the modern scientific understanding of this systemic equilibrium, illuminating how inflammatory status, disease states, and physiological transitions rapidly override baseline nutritional inputs.

In the paradigm of infection dynamics, host iron sequestration emerges as a cornerstone of nutritional immunity, systematically depriving invading pathogens of the essential trace element required for replication, biofilm structure, and virulence expression. However, this physiological defense mechanism inherently introduces a clinical paradox. The cellular hypoferremia driven by inflammation-induced hepcidin elevation concurrently impairs the proliferative capacity, metabolic competence, and functional maturation of lymphoid cells, notably CD8 + T lymphocytes and natural killer cells, potentially undermining long-term adaptive immune efficacy and vaccine responsiveness. Furthermore, this defensive locking mechanism can be leveraged by specialized intracellular pathogens, demonstrating that systemic iron manipulation holds divergent outcomes depending on the microbiological niche of the infectious agent.

Concurrently, conventional clinical strategies utilized to manage iron deficiency anemia within vulnerable cohorts—particularly patients suffering from inflammatory bowel diseases, celiac disease, or advanced age-related chronic inflammation—require a critical re-evaluation. The oral administration of traditional high-dose ferrous salts frequently overwhelms the absorptive capacity of the upper gastrointestinal tract, leaving a substantial fraction of unabsorbed labile iron in the colonic lumen. This unabsorbed pool catalyzes harmful Fenton reactions that generate tissue-damaging reactive oxygen species, compromises intestinal barrier integrity, and fosters a state of microbial dysbiosis that selectively promotes the proliferation of opportunistic, iron-seeking pathogens. While modern intravenous formulations with low labile iron content offer a robust mechanism to bypass the restricted gastrointestinal tract without accelerating systemic infectious phenotypes, clinical deployment must remain closely monitored against individual comorbidities, baseline tissue ferritin parameters, and current infection status.

In conclusion, achieving optimal clinical outcomes in the management of altered iron states demands a shift away from uniform supplementation models and toward highly personalized, pathophysiology-driven interventions. Future research must prioritize the development of next-generation oral formulations that possess enhanced targeted bioavailability, minimize the intestinal labile iron footprint, and protect the homeostatic architecture of the gut microbiota. Ultimately, deciphering the continuous molecular cross-talk between host iron status, nutritional variables, and pathogen survival tactics remains indispensable for designing highly refined therapeutic strategies capable of correcting hematological deficits while preserving robust antimicrobial defense systems.
